# A Health Literacy Intervention Targeting Chronic Kidney Disease Patients and Healthcare Professionals is Cost-saving: Findings from the Netherlands

**DOI:** 10.1007/s11606-025-09697-y

**Published:** 2025-07-08

**Authors:** Matheus S. Gurgel do Amaral, Marco D. Boonstra, Simon van der Pol, Ofer Engel, Gerjan J. Navis, Sijmen A. Reijneveld, Andrea F. de Winter

**Affiliations:** 1 University of Groningen, University Medical Center Groningen, Department of Health Sciences, Community and Occupational Medicine, Groningen Groningen, The Netherlands; 2 Health-Ecore, Zeist Utrecht, The Netherlands; 3 University of Groningen, University Medical Center Groningen, Department of Nephrology, Groningen Groningen, The Netherlands; 4 University Medical Center Groningen, Groningen, The Netherlands

**Keywords:** health literacy, chronic kidney disease, intervention, cost-effectiveness, hypertension

## Abstract

**Background:**

Chronic kidney disease (CKD) is a progressive disease with high societal costs. CKD progression is influenced by patients’ health literacy, i.e., the patients’ ability to deal with information related to their own health.

**Objective:**

To assess the cost-effectiveness of Grip on Your Kidneys (GoYK), a multi-component health literacy intervention for CKD patients and their healthcare professionals.

**Design and Main Measures:**

We performed a cost-utility analysis of the GoYK intervention, measuring health outcomes in terms of quality-adjusted life years (QALYs) and costs in euros, and extrapolated the results to a lifetime horizon using a Markov model.

**Subjects:**

The GoYK study was a quasi-experimental study involving 147 patients with CKD and 48 healthcare professionals from Dutch general practices and nephrology clinics, followed over nine months.

**Key Results:**

We found that the GoYK intervention would yield a cost-saving of €28,616 and a gain of 0.37 QALYs per CKD patient across the life course. Moreover, GoYK would avert 31 deaths and 113 new dialyses per 1,000 patients in the first 10 years. These results mainly stemmed from the intervention effect on the transition to end-stage renal disease, where reductions of as little as 1% would already mean GoYK is cost-saving. The intervention remained cost-effective in multiple sensitivity and scenario analyses.

**Conclusions:**

GoYK might be a solution to slow CKD progression and, thereby, reduce CKD costs. Given these promising findings and the novelty of our study, further research should confirm our findings before broad implementation.

**Supplementary Information:**

The online version contains supplementary material available at 10.1007/s11606-025-09697-y.

## INTRODUCTION

Chronic kidney disease (CKD) is a progressive disease with a high societal burden. CKD overlaps with and amplifies the effect of other chronic diseases, resulting in exponentially worse health outcomes and costs.^1,2^ These costs have been growing over the last decades, due to the increasing prevalence of CKD and the high cost of therapies.^3^ Consequently, CKD is among the most expensive diseases for healthcare systems, with a cost estimated at 140 billion euros annually in Europe.^4^ In the Netherlands, the average annual cost per patient can amount to 109,000 euros, when patients reach the more severe stages of the disease.^5^ Thus, it is imperative to prevent CKD progression, as even small improvements in renal function could represent large budget savings in the long term.^6,7^

Interventions that address low health literacy may provide a cost-effective solution for slowing CKD progression. Health literacy is the degree to which individuals have the capacity to obtain, process, and understand basic health information and services needed to make appropriate health decisions.^8^ Individuals with low health literacy have worse disease management skills, which have been shown to lead to faster CKD progression, higher mortality, and higher costs.^9,10^ Low health literacy also leads to less effective communication between patients and healthcare professionals, which has a negative influence on the quality of care.^11^ Therefore, interventions focused on health literacy might be a solution to improve CKD care and, thereby, mitigate CKD progression and CKD burden. Fortunately, such interventions are likely to be cost-effective, due to the minimal costs of this approach.^12–14^ To the best of our knowledge, however, health-economic studies of health-literacy-tailored interventions in CKD still do not exist.

We developed and tested the Grip on Your Kidneys (GoYK) intervention, a multi-component health literacy intervention for CKD patients and healthcare professionals.^15^ During a 9-month follow-up, when compared to care-as-usual, the group of patients that received the intervention showed improvements in levels of hypertension.^15^ Given that hypertension is associated with CKD,^16^ we hypothesized that the GoYK intervention may have a protective effect on CKD progression, thereby reducing costs. Thus, the present study aimed to investigate the cost-effectiveness of the GoYK intervention, compared to care-as-usual, via a reduction in CKD progression.

## METHODS

We performed a cost-utility analysis of the GoYK intervention, measuring health outcomes in terms of quality-adjusted life years (QALYs) and costs in euros, and extrapolated the results to a lifetime horizon using a Markov model. Details of the methods used are described in the following subsections.

### Cost-effectiveness Analysis

The cost-effectiveness analysis was performed according to the Dutch guidelines for economic evaluations in healthcare.^17^ The GoYK study followed patients and healthcare professionals for nine months, but the effect of the intervention on disease costs and quality of life is expected to increase with time and be more expressive later in life. Therefore, a Markov model was constructed to extrapolate the cost and effects of the GoYK intervention to a lifetime horizon, simulating outcomes until all patients had died.

### GoYK Effectiveness Study

GoYK was tested in a quasi-experimental study with 147 CKD patients and 48 healthcare professionals from Dutch general practices and nephrology clinics. The control group received only care-as-usual, while the intervention group also received the GoYK intervention. Patients in the intervention group were provided with simple, visually engaging information to enhance their disease knowledge and self-management. They also received a consultation card to improve communication with healthcare professionals during consultations. Healthcare professionals in the intervention group completed an e-learning course and participated in a workshop on health literacy. Findings based on data from questionnaires and patient records showed that, after 9 months, uncontrolled hypertension rates dropped in the intervention group (odds ratio = 0.45, 95% confidence interval (95%CI) [0.20–0.99]) and rates of lifestyle discussions increased (difference = 0.69, 95%CI [0.14–1.25]). Moreover, professionals in the intervention group used more health literacy strategies during consultations (difference = 0.56, 95%CI [0.19–0.93]).

### Model Structure

**Figure 1:**
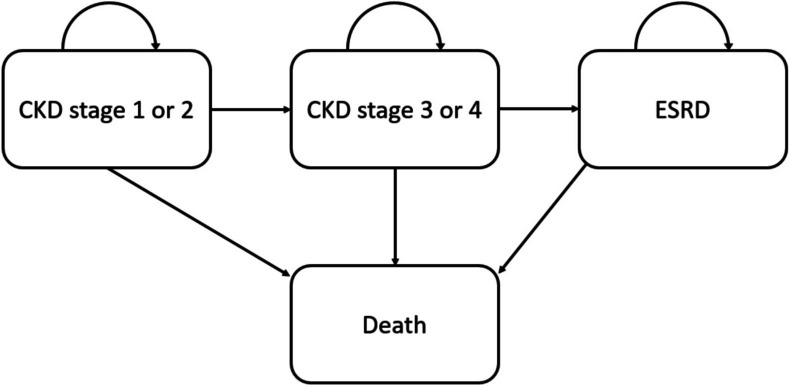
Markov model structure. CKD: chronic kidney disease, ESRD: end-stage renal disease.

In the Markov model (Fig. 1), a hypothetical group of 1,000 patients with CKD was followed, either receiving the GoYK intervention or care-as-usual. Patients could progress between four health states: CKD stages 1 or 2 (CKD 1/2), CKD stages 3 or 4 (CKD 3/4), end-stage renal disease (ESRD), and death. These health states mirror the organization of CKD care in the Netherlands, where care for CKD 1/2 is usually provided in primary care, CKD 3/4 in secondary care, and ESRD in dialysis centers. After each cycle, whose length was one year, patients could remain in the same CKD stage, progress to the next CKD stage, or die. We assumed that CKD patients progressed without skipping any stage and that they might die at any time. To match the characteristics of the population of the GoYK study, we used a starting age of 70, with 60% of males, and placed 7.3% of the patients in CKD 1/2 and 92.7% in CKD 3/4. Our analysis was performed from a societal perspective, meaning that it included all relevant societal costs and benefits. In line with Dutch guidelines, costs were discounted at an annual rate of 4% whereas health benefits were discounted at an annual rate of 1.5%.^17–19^ The Markov model was constructed using Microsoft Excel 2016 and it was clinically and technically validated by specialists in the areas of Nephrology and Health Economics.

### Model Parameters

The main parameters used in the model regarded the transition probabilities to subsequent CKD stages, mortality, quality of life, and costs.

#### Transition Probabilities and Mortality

CKD transition probabilities for the control group were retrieved from the literature (Table 1). For the intervention group, these probabilities were multiplied by the effect of GoYK on CKD progression. We estimated this effect by synthesizing the effect of GoYK on hypertension (from the GoYK study) and the effect of hypertension on CKD progression (from medical literature). More details on this estimation can be found in the Supplementary Methods. We assumed the effect of the intervention would decrease by 12.5% per year,^20^ but return to its full effect every five years, when the intervention would be repeated. The effect of GoYK on mortality was derived from published mortality risks. We used Dutch age-dependent all-cause mortality in the general population for CKD 1/2,^21^ and multiplied it by the relative mortality risk to obtain the mortality risk for CKD 3/4.^21–23^ The same was done to obtain mortality risks for ESRD, but the ESRD relative mortality risk was adjusted based on age, given that the differences in mortality risk between ESRD and the general population vary with age.^24^ The impact of these estimations was investigated in sensitivity analyses. All input data included in the model can be found in Table 1.

**Table 1:** Input Parameters Used in the Markov Model

Parameter	Value	95% CI	Distribution	Source
*Mortality*				
Incident mortality CKD 1/2	Age-dependent	0.01 to 0.39*	beta	^(21)^
Incident mortality CKD 3/4	Age-dependent	0.03 to 0.70*	beta	^(22)^
Incident mortality ESRD	Age-dependent	0.11 to 1.00*	beta	^(21,23)^
*Annual transition probabilities*				
CKD 1/2 to CKD 3/4	0.10	0.04 to 0.12*	beta	^(47–49)^
CKD 3/4 to ESRD	0.09	0.03 to 0.17*	beta	^(49,50)^
*Quality of life (utilities)*				
CKD 1/2	0.93	0.81 to 1.00	beta	^(25)^
CKD 3/4	0.80	0.68 to 0.98	beta	Derived
ESRD	0.56	0.49 to 0.62	beta	^(26)^
*Intervention effect (relative risk)*				
Transition CKD 1/2 to CKD 3/4	0.81	0.71 to 0.92	normal	Association GoYK and hypertension: derived Association hypertension and CKD: ^(51)^
Transition CKD 3/4 to ESRD	0.73	0.65 to 0.82	normal	Association GoYK and hypertension: derived Association hypertension and ESRD:^(52)^
*Annual costs (euros)*				
Direct medical costs				
CKD 1/2	1,844	1,475 to 2,213	gamma	Prevalence: ^(21,48,53,54)^ Costs: ^(37)^
CKD 3/4	2,394	1,915 to 2,873	gamma	Prevalence: derived Costs: ^(37)^
ESRD	98,221	54,666 to 141,775	gamma	Costs: ^(5)^
Indirect medical costs				
Last year of life	age- and sex-dependent	43,353 to 54,770	gamma	^(29)^
Other years of life	age- and sex-dependent	5,415 to 38,829	gamma	^(29)^

CKD: chronic kidney disease; ESRD: end-stage renal disease; CI: confidence interval; Derived: obtained from the data of the GoYK study; *Total range reported in place of 95%CI

#### Quality of Life (Utilities)

We assigned utility indexes, in QALYs, to each CKD stage (Table 1). The effect of GoYK on QALY gains was based on the difference in CKD-stage distribution induced by the intervention. CKD 3/4 was assigned a utility index based on the results of the baseline EQ-5D questionnaire applied to participants during the GoYK study. Given that the GoYK study did not assess ESRD patients and had too few patients in CKD 1/2, the utility scores for these stages were obtained from the literature.^25,26^

#### Costs

Direct medical costs for ESRD were obtained from literature,^5^ assuming all patients in ESRD need hemodialysis. The costs specific to CKD 1/2 and CKD 3/4 have not been evaluated in the Netherlands, so we calculated them based on general non-dialysis CKD costs and on the costs of the main CKD treatment targets in the Netherlands,^27^ namely diabetes, hypertension, and cardiovascular disease. The difference in costs between CKD 1/2 and CKD 3/4 corresponded, therefore, to the difference in the prevalence of those treatment targets in each CKD stage. The prevalence of each disease in CKD 3/4 was derived from the GoYK study. The prevalence for CKD 1/2 could not be derived from the GoYK study, given that it only included a few participants in these stages, so we used data from the general population registered in GP practices in the Netherlands.^21^ This calculation led to costs comparable to those from neighboring countries.^28^ Indirect medical costs were obtained from a Dutch registry^29^ and included as a sensitivity analysis. Non-medical costs, collected in the GoYK study, did not differ between groups and were not included in the Markov model.

GoYK costs regarded the development, implementation, and maintenance of the intervention. They regarded the development of digital and paper material, printing fees, website domain license, workshops for healthcare professionals, promotion of the intervention, and the hiring of a GoYK manager and educator, who would be responsible for periodically updating the intervention, giving workshops, managing marketing, and other administrative functions related to the implementation and maintenance of the intervention. We assumed the workshops with healthcare professionals would be repeated every five years after the beginning of the study. We did not include the cost of the work hours lost by healthcare professionals during the workshops, because we assumed they could receive accreditation points. These costs were, however, included as a sensitivity analysis. All costs were expressed in 2021 euros (Table 1), indexed according to the Dutch consumer price index.^30^

### Sensitivity and Scenario Analyses

To assess the sensitivity of the results to changes in model assumptions and inputs, we performed sensitivity and scenario analyses.

In the probabilistic sensitivity analysis, all parameters presented in Table 1 were varied within their 95%CI in 10,000 iterations,^31^ based on the pre-specified statistic distributions shown in Table 1. Results of the probabilistic sensitivity analysis are presented in a cost-effectiveness plane.

In the univariate sensitivity analyses, we repeated the main analysis using the upper or lower bound of the 95%CI of the parameters in Table 1, to assess their individual influence on the results. The univariate sensitivity analyses are presented in a tornado diagram, showing the most influential parameters on top of the graph.

In the scenario analyses, we assessed how changing various model variables would affect the results, which were considered cost-effective when the incremental cost-effectiveness ratio (ICER) was below 80,000 euros/QALY.^32,33^ Among the scenario analyses, we included a sample compatible with the general CKD population in the Netherlands, with a lower starting age of 55 years and a suitable distribution of patients in CKD 1/2 and CKD 3/4 stages.^34^ Finally, given that the effect on ESRD development was the intervention’s most influential parameter in the univariate sensitivity analyses, we assessed the effect of multiple variations of this parameter on the results.

## RESULTS

### Cost-effectiveness

The total costs per patient for the GoYK intervention and care-as-usual were €188,136 and €216,752, respectively, within the lifetime horizon. In the same period, total QALYs per patient for the GoYK intervention and care-as-usual were 7.28 and 6.91, respectively. This reflects an expected cost-saving of €28,616 and a gain of 0.37 QALYs per patient who received the GoYK intervention.

The effects of the GoYK intervention on costs and QALYs were most evident in the first years, mainly due to the retardation of progression from CKD 3/4 to ESRD (Supplementary Figures S1a and S1b). In the first 10 years, the intervention averted 31 deaths and 113 new dialyses per 1,000 patients. Around 55% of the patients were deceased after 10 years in the intervention and control groups. This percentage increased to 90% after 17 years and 99% after 22 years.

### Sensitivity and Scenario Analyses

**Figure 2:**
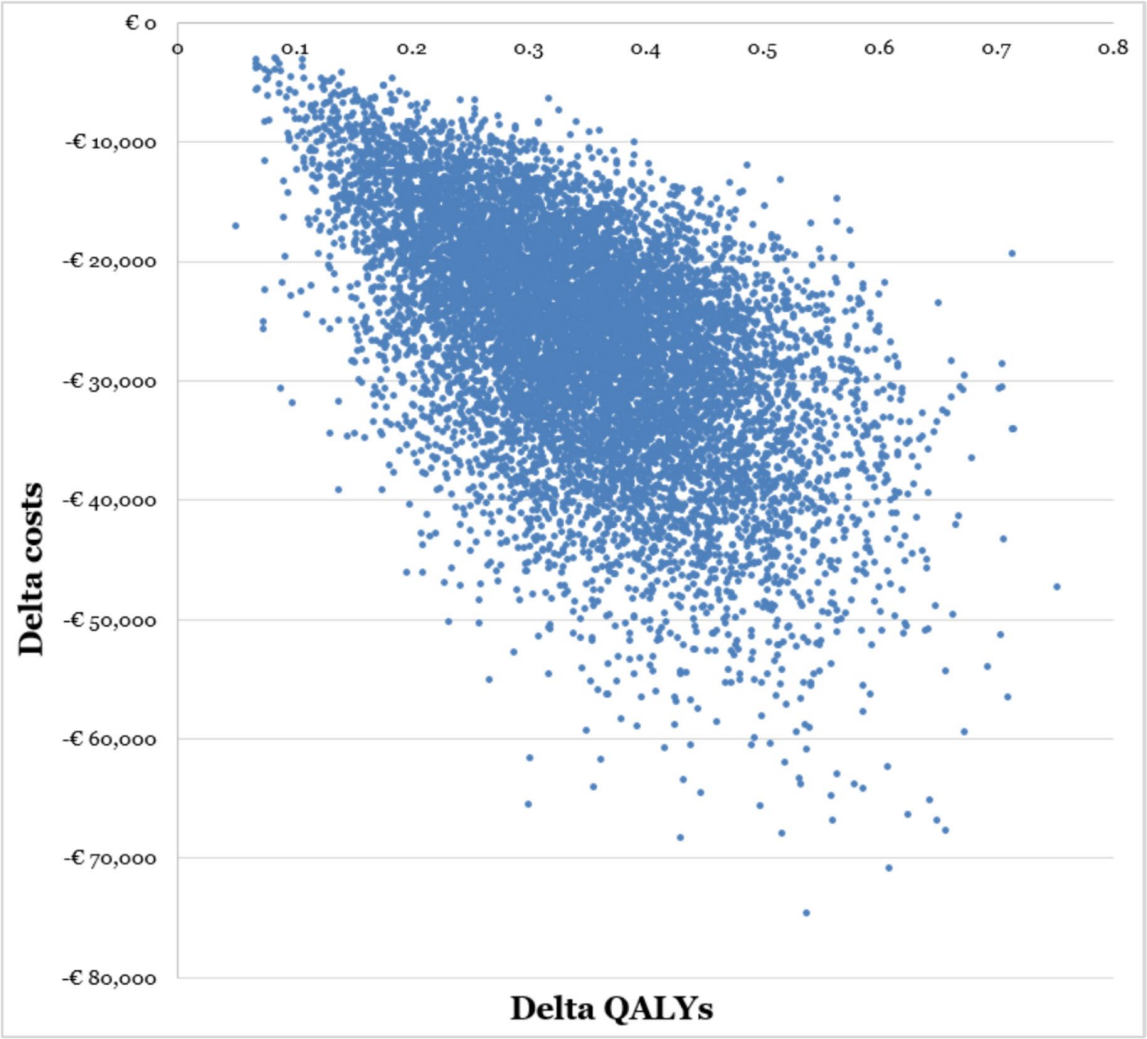
Cost-effectiveness plane. QALY: quality-adjusted life year.

Probabilistic sensitivity analyses revealed that all the 10,000 simulations performed resulted in cost savings for the GoYK intervention. This is illustrated in Fig. 2, where all simulations fell within the bottom right quadrant of the cost-effectiveness plane.

**Figure 3:**
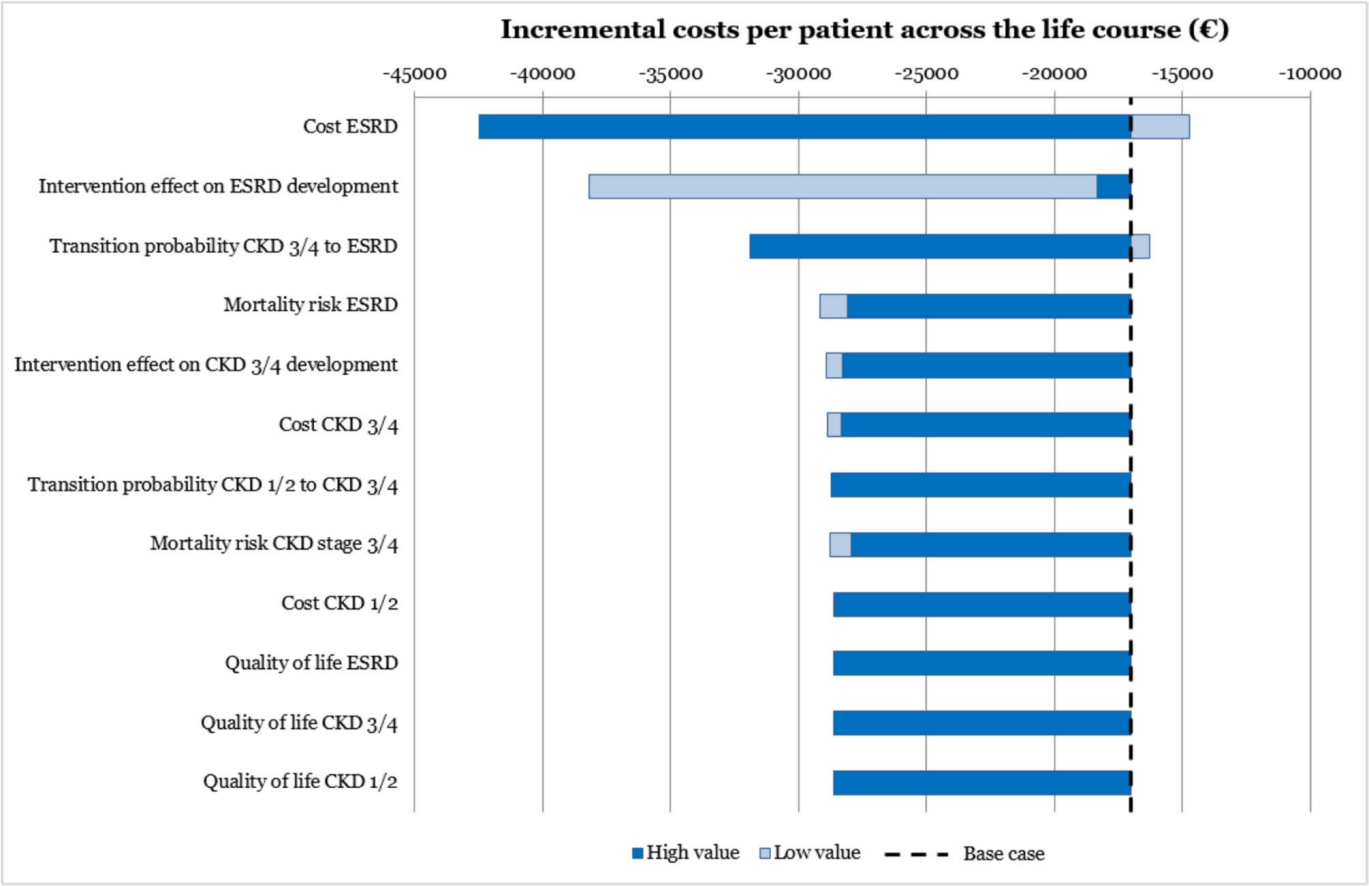
Univariate sensitivity analyses. ESRD: end-stage renal disease; CKD: chronic kidney disease.

Univariate sensitivity analyses (Fig. 3) showed that the model was most sensitive to the cost of ESRD and the effect of the intervention on the transition from CKD 3/4 to ESRD. The quality of life in CKD 1/2 and CKD 3/4 were the least influential parameters. Regardless of the parameters analyzed, the GoYK intervention remained cost-saving.

**Figure 4:**
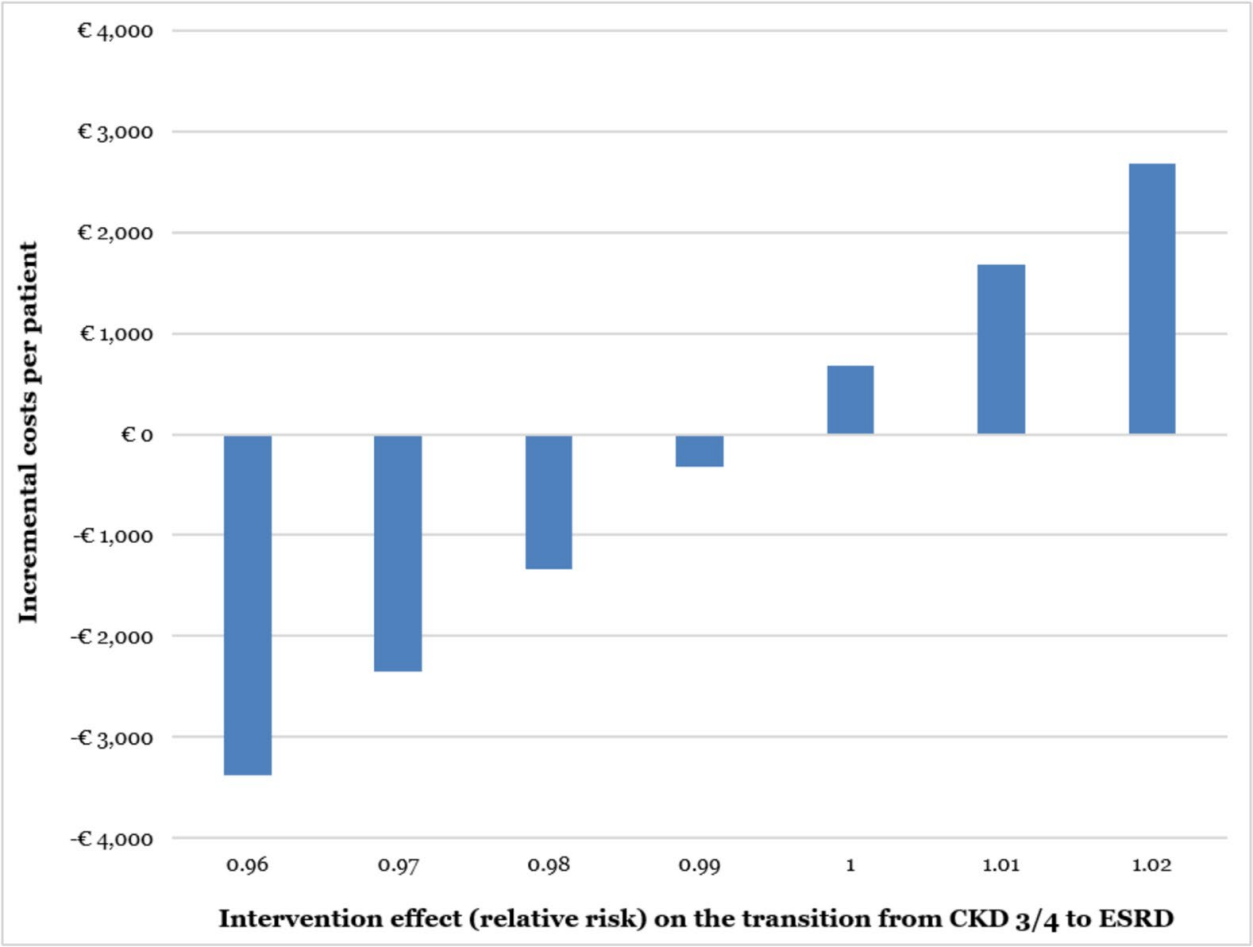
Variations of the effect of the intervention on the transition from CKD 3/4 to ESRD. CKD: chronic Kidney Disease; ESRD: end-stage renal disease.

The scenario analysis (Table 2) showed that the GoYK intervention remained cost-effective for different time horizons, intervention effects, sample distributions, starting age, mortality risk, and extra costs. The assessment of multiple variations of the intervention effect (Fig. 4) showed that GoYK would be cost-saving if it promoted at least a 1% reduction in the transition between CKD 3/4 and ESRD.

**Table 2:** Scenario Analyses

	Incremental costs^*^	Incremental QALYs^*^	**ICER**^†^
**Base case**^‡^	−28,616	0.37	CS
**Other scenarios**			
Time horizon			
5 years	−14,670	0.07	CS
10 years	−26,042	0.20	CS
Starting sample distribution			
100% CKD 1/2	−18,188	0.44	CS
100% CKD 3/4	−29,437	0.37	CS
Starting age			
55 years old^§^	−45,509	0.81	CS
Mortality risk			
Constant for all stages^║^	−75,102	0.17	CS
Extra costs			
Indirect medical costs	−25,682	0.37	CS
Work hours lost by professionals	−28,611	0.37	CS
Intervention effect			
Only in the first year	19,589	0.74	26,530
Constant over time	−37,112	0.48	CS
No effect on transition to CKD 3/4	−28,081	0.35	CS
No effect on transition to ESRD	683	0.02	31,116
Non-discounted costs and QALYs	−35,718	0.43	CS

*QALY: Quality-adjusted life year; ICER: Incremental cost-effectiveness ratio (in euros/QALY); CS: cost-saving; CKD 1/2: Chronic kidney disease stages 1 or 2; CKD 3/4: Chronic kidney disease stages 3 or 4*

^*^ Costs and QALYs per patient over the lifetime, unless otherwise defined

^†^* Considering a willingness-to-pay threshold of €80,000/QALY gained*

^‡^* Parameters of base case: lifetime horizon, percentage of sample starting in CKD 1/2* = *7.3%, starting age* = *70 years, indirect medical costs and work hours lost by professionals not included, intervention effect decreases by 12.5% every year, costs and QALYs discounted at annual rates of 4% and 1.5%, respectively*

^§^* Percentage of sample starting in CKD 1/2* = *59.3%*

^║^* Equal to Dutch age-dependent all-cause mortality*

## DISCUSSION

Our study showed that the GoYK intervention would yield substantial cost-savings across the life course of CKD patients, as well as a substantial reduction in deaths and in the need for dialysis in the first years. GoYK is likely to yield cost-savings due to its simple and inexpensive implementation and maintenance, along with its expected impact on the reduction of CKD progression, through improvements in hypertension control.

The biggest impact of GoYK on costs and the number of deaths stems from the reduction in the transition to ESRD, where renal replacement therapies are required. Given that these therapies are extremely expensive and burdening, even small decreases in the number of patients that need them would result in large gains in costs and quality of life. Moreover, since the mortality rate of ESRD is considerably higher than that of other CKD stages, a reduction in ESRD would also mean a reduction in total deaths. Our findings corroborate the results of previous studies on CKD, which showed that the effectiveness of treatments and educational programs in reducing the progression to ESRD is an important factor in making an intervention cost-saving.^35,36^ Our results may still be an underestimation of the cost-saving of GoYK, given that most of the tools and skills covered by the intervention are not specific to CKD, potentially covering other diseases and leading to even greater gains. One relevant example is cardiovascular disease, a burdening and expensive condition that is closely linked to CKD.^16,37^

GoYK remained cost-effective in all the sensitivity analyses performed, demonstrating the robustness of our results. Almost all sensitivity analyses led to cost-saving results, and the ones that were not cost-saving were still cost-effective, remaining below the Dutch cost-effectiveness threshold of €80,000, recommended for diseases with a high burden like CKD.^32,33^ However, it is worth mentioning that GoYK might reach a higher number of patients than used in our analysis, which would translate into a proportionally lower intervention cost per patient. We explored this possibility in an additional analysis, revealing that even if GoYK had no effect on ESRD development, extending it to a minimum of 1,250 patients would result in an ICER below €20,000 per QALY. This is the lowest threshold recommended by the Dutch guidelines, normally applied to diseases with a burden much inferior to CKD.

### Strengths and Limitations

Our study had several strengths. We used data from the GoYK study, which included real-life data of patients and healthcare professionals from multiple organizations in primary and secondary care organizations. This comprehensive sample closely mirrored the complexity and diversity of Dutch CKD healthcare. Moreover, we were the first ones to study the cost-effectiveness of an intervention focused on health literacy, an important social determinant of health with notorious negative impacts on chronic diseases, in the CKD setting.

Our study also had limitations. First, the effect of GoYK on CKD progression was not assessed directly but estimated using an intermediate outcome, namely hypertension. This increased the uncertainty of our findings, as indirect estimations may fail to account for non-observed variables that might influence the associations, and because they are contingent on model assumptions and limitations. Nevertheless, we employed conservative assumptions when performing the calculation. It is also worth noting that the use of indirect estimation often gives a reliable estimation of the final outcome, the reason why it is commonly used in cost-effectiveness studies.^31,38,39^ Second, we analyzed the effect of GoYK on CKD progression via one mediator only and did not take into account other related outcomes, such as cardiovascular diseases.^40–42^ This approach could have led to an underestimation of the real health gains. Third, given that the majority of the individuals in GoYK were older and therefore retired, we could not collect information on non-medical costs, such as work absenteeism. This may have underestimated our findings, given that more advanced stages of CKD are related to higher absenteeism.^43^ Finally, we assumed all ESRD patients received dialysis, whereas around 13% would receive renal transplantation, a treatment that can be less expensive in the long term.^23^ This could have resulted in an overestimation of the cost savings.

#### Implications

We suggest that healthcare organizations ultimately implement GoYK, given its substantial potential for saving costs. However, as this is the first study to assess the cost-effectiveness of a health literacy intervention in CKD, further research should confirm its findings before broad implementation.

To achieve implementation, GoYK needs to be incorporated into health guidelines and reimbursement schedules by healthcare financers. GoYK can then be easily implemented, with a minimum burden for patients and professionals, since it has been designed to be integrated into standard CKD care. Interventions such as GoYK are extremely important given that initiatives focused on the prevention of CKD progression are often scattered, inadequate, and incongruent, despite their potential for reducing disease burden and societal costs.^2,44^ Thus, GoYK represents a means to target social determinants of health and promote disease prevention, with consequent improvements in health.^45,46^

Given the uncertainties present in our analysis, the implementation of the intervention should be combined with research on the intervention’s effect for a longer period in a larger number of individuals. That could enable a better estimation of the effect of the intervention on clinical outcomes, such as eGFR and health behaviors, and the identification of subgroups that could benefit most from the intervention, e.g. patients recently diagnosed with CKD.

Moreover, the (cost-)effectiveness of such a multi-component health literacy intervention should be tested in other countries, to assess if it is suitable for other cultures and healthcare system configurations. Finally, as GoYK covers skills that are also useful in the management of other chronic diseases, similar interventions could be tested for other conditions, such as cardiovascular disease, COPD, and cancer.

## CONCLUSION

GoYK, a multi-component health literacy intervention for CKD patients and healthcare professionals, seems to lead to large cost-savings across the life course of CKD patients, and to a substantial reduction in mortality and need for dialysis, compared to care-as-usual. We recommend the implementation of GoYK by healthcare organizations and its incorporation into health guidelines and reimbursement schedules. Given the novelty of our study, the implementation of GoYK should be combined with further research on its long-term effects and subgroups that could benefit the most from it.

## Supplementary Information

Below is the link to the electronic supplementary material.

Supplementary file1 (PDF 439 KB)

## Data Availability

The datasets analyzed during the current study are available from the corresponding author on reasonable request.
