# Supplementary material for: A Health Literacy Intervention Targeting Chronic Kidney Disease Patients and Healthcare Professionals is Cost-saving: Findings from the Netherlands
Source: J Gen Intern Med. 2025 Jul 8;41(4):967–75. doi: 10.1007/s11606-025-09697-y (PMC13009444; doi:10.1007/s11606-025-09697-y)
Supplement: Supplementary file 1 — Supplementary file1 (PDF 439 KB) [file 11606_2025_9697_MOESM1_ESM.pdf]

Supplementary Appendix for Gurgel do Amaral MS, Boonstra MD, Van der Pol S, Engel O, Navis GJ, Reijneveld SA, De Winter AF: *A Health Literacy Intervention Targeting Both Chronic Kidney Disease Patients And Healthcare Professionals Is Cost-Saving: Findings From The Netherlands*.

TABLE OF CONTENTS

Supplementary Methods Detailed description of the calculation of the effect of GoYK on CKD progression, via hypertension……………………………………………………………………………………………………2

Supplementary Figure S1a Average costs per patient accumulated during the life course……………………8

Supplementary Figure S1b Average QALYs per patient accumulated during the life course…………………..8

Supplementary Methods

Detailed description of the calculation of the effect of GoYK on CKD progression, via hypertension

A. Rationale

The Grip on Your Kidneys (GoYK) study assessed the effects of the intervention on hypertension and not directly on chronic kidney disease (CKD) progression. Therefore, we estimated the effect of GoYK on CKD progression by synthesizing the data of the GoYK study with data from the literature. Below, you can find the schematic summary of the model created for the calculation of the effect of GoYK on CKD progression:

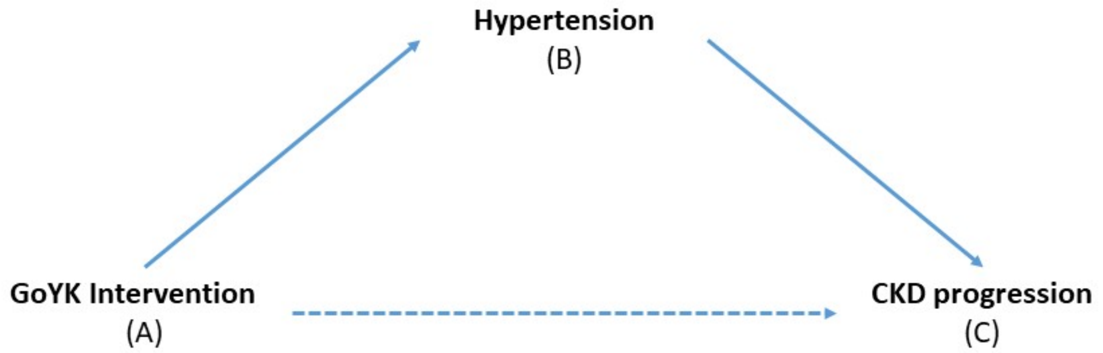

Figure SM1: Schematic summary of the calculation of the effect of GoYK on CKD progression

In the figure above, A is the GoYK intervention (A=1 is intervention and A=0 is control), B is hypertension (B=1 is high blood pressure and B=0 is normal blood pressure), and C could be the transition from CKD 1/2 to CKD 3/4, or the transition from CKD 3/4 to ESRD (C=1 is the transition to next CKD stage and C=0 is remaining in the same stage). The association $A \rightarrow B$ was derived from the GoYK study, the associations $B \rightarrow C$ were retrieved from articles from the medical literature, and the association $A \rightarrow C$ was calculated by us. In this study, we focus only on one part of the intervention's effect on the outcome, namely the part mediated by hypertension. Below, you can find the effects reported in each study and the total mediated effect calculated by us:

Table SM1: Summary of the parameters used in the model to calculate the effect of GoYK on CKD progression

**Variables of the model**		**Effects of individual studies**		**Total mediated effect**
Mediator (B)	Final outcome (C)	OR (A → B)	HR (B → C)	RR (A → C)
Hypertension	CKD 3/4	0.45 [0.20-0.99][S1]	1.83 [1.34-2.48][S2]	0.81 [0.71-0.91]
	ESRD	0.45 [0.20-0.99][S1]	2.28 [1.71-3.02][S3]	0.73 [0.65-0.82]

OR: odds ratio of the effect of GoYK (A) on hypertension (B)
HR: hazard ratio of the effect of hypertension (B) on CKD progression (C)
RR: relative risk of the effect of the GoYK intervention (A) on CKD progression (C)

Formally, we focused on the effect of the intervention (A) on the outcome (C), which is independently conditional on hypertension (B):

$$A \rightarrow B \rightarrow C$$
$$A \perp C \mid B$$

The resulting risk ratio would provide an upper limit on the relative risk of the outcome when comparing the two treatment groups. In other words, any residual effect of the intervention on the outcome that does not involve the status of hypertension would further decrease the relative risk. Assuming $A \perp C|B$, we can show that:

$$P(C|A) \;=\; P(C|B)P(B|A) \;+\; P(C|\bar{B})P(\bar{B}|A)$$

The relative risk $RR_{C,A}$ is therefore given in formula 1:

$$RR_{C,A} = \frac{P(C|A)}{P(C|\bar{A})} = \frac{RR_{C,B} \cdot P(B|A) + P(\bar{B}|A)}{RR_{C,B} \cdot P(B|\bar{A}) + P(\bar{B}|\bar{A})} \quad (1)$$

To calculate this $RR_{C,A}$, we need the conditional probabilities for the transition $A \rightarrow B$ and the risk ratio for the transition $B \rightarrow C$. For the calculation of $RR_{C,B}$, we used studies from the literature.[S2,S3] Both studies reported their results in terms of hazard ratios and not risk ratios, so we sought a way to estimate the risk ratios from the hazard ratios.

The empirical research literature often treats hazard ratios as risk ratios, an interpretation that holds approximately when the outcome is rare. However, VanderWeele offers a more accurate approximation when an upper and lower limit can be determined for the probability of the incidence in both study arms.[S4] In this case, the optimal minimax transformation of the hazard ratio can be shown to approximate the risk ratio under a proportional hazard model where ϕ is the hazard ratio and p_t, p_c are the unknown probability of incidence for the treatment and the control group, respectively; both probabilities bounded by known limits w, v . If $\phi > 1$ and $w < p_c < p_t < u$, the optimal bias-ratio minimax conversion on the interval $[w, u]$ of a hazard ratio ϕ is given by the following square-root transformation:

$$\left\{\frac{1-(1-w)^{\phi}}{1-(1-u)^{1/\phi}}\frac{u}{w}\right\}^{1/2} \quad (2)$$

Since this expression is an approximation, applying it directly to the lower and upper bounds of the hazard ratio confidence interval would not yield a 95% coverage over repeated samples of the true risk ratio. However, using a numeric grid search for the outcome probabilities p_t, pc within a specified interval, it is possible to determine the maximum bias ratio for the approximation, and to use it to obtain a conservative confidence interval.

B. Estimating the effect of the intervention on the transition from CKD 1/2 to CKD 3/4

Kanno et al report that the hazard ratio for the progression to CKD 3/4 over a period of 10 years, comparing individuals with and without hypertension, is 1.83 $[1.34,\ 2.48]$; the model was fully adjusted for age, sex, smoking, drinking, obesity, cardiovascular disease, diabetes mellitus, hypercholesterolemia, anti-hypertensive treatment, baseline eGFR, number of follow-up examinations, and year of baseline examination.[S2] The outcome probabilities in this study are bounded by the interval $[0.015{,}0.30]$.

Applying the approximation in (2) to the results from Kanno et al, the optimal bias-ratio minimax conversion for the hazard ratio of 1.83 is approximately estimated as a risk ratio of 1.76, and the lower and upper limit of the hazard ratio confidence interval can be similarly transformed to obtain a risk ratio interval of $[1.31{,}2.34]$. Since these are biased approximations of the limits of the risk ratios, we need to correct these limits by estimating the maximum bias associated with this approximation, a correction that secures 95% coverage over repeated samples of the true risk ratio.

A numeric grid search shows that for outcome probabilities in the range $[0.015{,}0.30]$, the bias ratio is always less than 5.5%. Hence, a confidence interval will be sure to have at least 95% coverage of the true risk ratio, provided that the square-root transformation of the lower limit of the hazard ratio confidence interval is divided by 1.055 and the square-root transformation of the upper limit of the hazard ratio confidence interval is multiplied by 1.055.

We therefore estimate that the hazard ratio of 1.83 $[1.34,\ 2.48]$ can be transformed to a risk ratio of 1.76 and a 95% confidence interval of $[1.24,\ 2.47]$.

Given this information, we can use the formula (1) above to estimate the risk ratio $RR_{C,A} = 0.81$, with a 95% confidence interval of [0.71, 0.92].

The calculations are demonstrated in the R script below:

```
#############################################
## Effect of intervention on hypertension ##
###########################################
num_treat <- 149 + 523
pb_a_treat   <- 149 / (149 + 523)  # p(b|intervention)
num_ctrl <- 361 + 256
pb_a_ctrl  <- 361 / (361 + 256)  # p(b|~intervention)




#####################
## Functions used ##
###################

cumulative_p <- function( p, n ) {
  1 - (1 - p)^n
}
```

```r
rr_from_hr <- function( w, u, phi ) {

  n <- length(phi)
  w <- rep(w, n)
  u <- rep(u, n)
  v <- w
  w <- if_else(phi < 1, u, w)
  u <- if_else(phi < 1, v, u)

  a <- 1 - (1 - w)^phi
  b <- 1 - (1 - u)^(1 / phi)
  (a / b * u / w)^(1/2)
}


max.bias <- function(w, u, phi, step = 0.05) {
  expand.grid(
    p1 = seq(w, u, step),
    p2 = seq(w, u, step)
    ) |>
    mutate(
      phi = log(1 - p1) / log(1 - p2),
      rr   = p1 / p2,
      rr.est = rr_from_hr(w, u, phi),
      bias = max(
        rr.est / rr,
        rr / rr.est
        )
      )
}


rr_c_a <- function(pb_a_t, pb_a_c, rr_c_b) {
  num   <- rr_c_b * pb_a_t + (1 - pb_a_t)
  denom <- rr_c_b * pb_a_c + (1 - pb_a_c)
  num / denom
}


get_ca_ci <- function(lb_rr_bc, ub_rr_bc) {

  rr_cb.sample <- runif(1, lb_rr_bc, ub_rr_bc)
  pb_a_treat.sample <- rbinom(1, num_treat, pb_a_treat) /
    num_treat
  pb_a_ctrl.sample <- rbinom(1, num_ctrl, pb_a_ctrl) /
    num_ctrl

  rr_c_a(pb_a_treat.sample, pb_a_ctrl.sample, rr_cb.sample)
}
```

```r
######################################################
## Effect of intervention on transition to CKD 3/4 ##
####################################################

# Hypertension --> CKD 3/4
# 10 years incidence probability (Kanno et al 2012)
# HR 1.83 [1.34 to 2.48]
pt.10  <-  109  / 386 # exposure (hypertension)
pc.10  <-  97   / 586  # control (no hypertension)

# 1 year incidence probability
pt.1 <- cumulative_p(pt.10, 1/10)
pc.1 <- cumulative_p(pc.10, 1/10)

rr_estimate <- rr_from_hr(.015, .30, 1.83) # 1.755239
max.bias(.015, .30, 1.83) |>
  pull(bias) |> max() # 1.055135

# lower bound
lb_max_bias <- max.bias(.015, .30, 1.34) |>
  pull(bias) |> max() # 1.055135
(rr_bc_lb <- rr_from_hr(.015, .30, 1.34)) # 1.309881 biased approximation
lb_rr_bc <- rr_bc_lb / lb_max_bias        # 1.241434 correction for bias

# upper bound
ub_max_bias <- max.bias(.015, .30, 2.48) |>
  pull(bias) |> max() # 1.055135
(rr_bc_ub <- rr_from_hr(.015, .30, 2.48)) # 2.343614 biased approximation
ub_rr_bc <- rr_bc_ub * ub_max_bias        # 2.472829 correction for bias

# Estimation of the main effect of the intervention
rr_c.a <- rr_c_a(pb_a_treat, pb_a_ctrl, rr_estimate)
# 0.8096753

# Estimation of confidence interval
df <- map_dbl(1:10000, \(x) get_ca_ci(lb_rr_bc, ub_rr_bc))
quantile(df, probs = c(0.025, 0.50, 0.975))
# 0.7055800 0.7949278 0.9162919
```

C. Estimating the effect of the intervention on the transition from CKD 3/4 to ESRD

We calculate the risk ratio of progression to ESRD just as above, using the hazard ratio reported by Kim et al; the model was fully adjusted for age, sex, smoking, alcohol consumption, exercise, low-income status, metabolic syndrome, proteinuria, and history of dyslipidemia, diabetes, and CKD.[S3] In this case, since the probability for the outcome (C) over the four years of follow-up is not more than 0.3%, the bias of the approximation in formula (2) is negligible, and we obtain an estimated risk ratio of 2.28 [1.71, 3.04]. Using the formula (1) to calculate the risk ratio of the intervention (A) on ESRD (C), we estimate the risk ratio to be 0.73 [0.65, 0.82]

The calculations are demonstrated in the R script below:

```r
####################################################
## Effect of intervention on transition to ESRD ##
##################################################

# Hypertension --> ESRD
# 4 years incidence probability (Kim et al 2021)
# HR 2.28 [1.71 3.04]
pt.4  <- 57 / 20868 # exposure (hypertension)
pc.4  <- 1053 / 1664488 # control (no hypertension)

# 1 year incidence probability
pt.1 <- cumulative_p(pt.4, 1 / 4)
pc.1 <- cumulative_p(pc.4, 1 / 4)

rr_estimate <- rr_from_hr(0.0002, .003, 2.28) # 2.278893
max.bias(0.0002, .003, 2.28, step = .0001) |>
  pull(bias) |> max() # 1.000413

# lower bound
lb_max_bias <- max.bias(0.0002, .003, 1.71, step = .0001) |>
  pull(bias) |> max() # 1.000413
(rr_bc_lb <- rr_from_hr(0.0002, .003, 1.71)) # 1.709406
lb_rr_bc <- rr_bc_lb / lb_max_bias

# upper bound
ub_max_bias <- max.bias(0.0002, .003, 3.04, step = .0001)  |>
  pull(bias) |> max() # 1.000413
(rr_bc_ub <- rr_from_hr(0.0002, .003, 3.04)) # 3.038159
ub_rr_bc <- rr_bc_ub * ub_max_bias

# Estimation of the main effect of the intervention
rr_c.a <- rr_c_a(pb_a_treat, pb_a_ctrl, rr_estimate)
# 0.7341925

# Estimation of confidence interval
df <- map_dbl(1:10000, \(x) get_ca_ci(lb_rr_bc, ub_rr_bc))
quantile(df, probs = c(0.025, 0.50, 0.975))
# 0.6502432 0.7257125 0.8162945
```

References:

S1. Boonstra MD, Gurgel do Amaral M, Navis G, et al. Effectiveness of a health literacy intervention targeting both chronic kidney disease patients and health care professionals in primary and secondary care: a quasi-experimental study. *J Nephrol*. 2024;37(9):2621-2633.

S2. Kanno A, Kikuya M, Ohkubo T, et al. Pre-hypertension as a significant predictor of chronic kidney disease in a general population: the Ohasama Study. *Nephrol Dial Transplant*. 2012;27(8):3218-23.

S3. Kim CS, Kim B, Choi HS, et al. Cumulative hypertension burden and risk of end-stage renal disease. *Hypertens Res*. 2021;44(12):1652-61.

S4. VanderWeele TJ. Optimal approximate conversions of odds ratios and hazard ratios to risk ratios. *Biometrics*. 2020;76(3):746-52.

Supplementary Figures

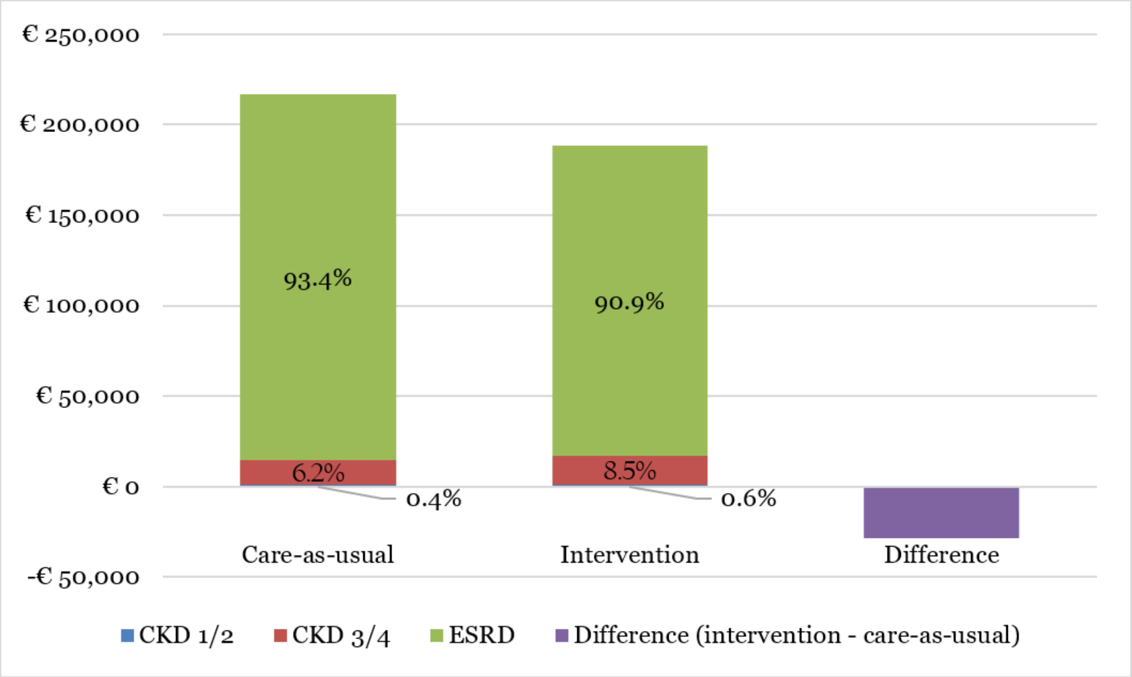

Figure S1a Average costs per patient accumulated during the life course

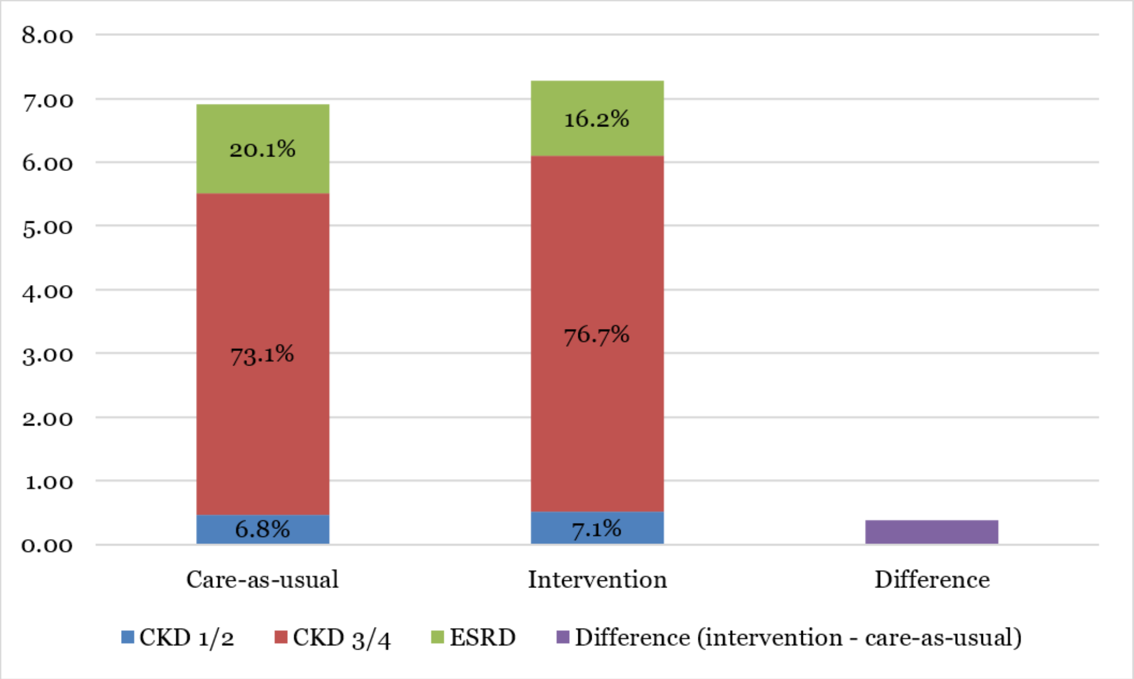

Figure S1b Average QALYs per patient accumulated during the life course
